# Two Tickets to Paradise: Multiple Dispersal Events in the Founding of Hoary Bat Populations in Hawai'i

**DOI:** 10.1371/journal.pone.0127912

**Published:** 2015-06-17

**Authors:** Amy L. Russell, Corinna A. Pinzari, Maarten J. Vonhof, Kevin J. Olival, Frank J. Bonaccorso

**Affiliations:** 1 Department of Biology, Grand Valley State University, Allendale, Michigan, United States of America; 2 Hawai'i Cooperative Studies Unit, University of Hawai'i at Hilo, Hawai'i National Park, Hawai'i, United States of America; 3 Department of Biological Sciences, Western Michigan University, Kalamazoo, Michigan, United States of America; 4 Environmental and Sustainability Studies Program, Western Michigan University, Kalamazoo, Michigan, United States of America; 5 EcoHealth Alliance, New York, New York, United States of America; 6 U.S. Geological Survey, Hawai'i National Park, Hawai'i, United States of America; CSIRO, AUSTRALIA

## Abstract

The Hawaiian islands are an extremely isolated oceanic archipelago, and their fauna has long served as models of dispersal in island biogeography. While molecular data have recently been applied to investigate the timing and origin of dispersal events for several animal groups including birds, insects, and snails, these questions have been largely unaddressed in Hawai'i’s only native terrestrial mammal, the Hawaiian hoary bat, *Lasiurus cinereus semotus*. Here, we use molecular data to test the hypotheses that (1) Hawaiian *L*. *c*. *semotus* originated via dispersal from North American populations of *L*. *c*. *cinereus* rather than from South American *L*. *c*. *villosissimus*, and (2) modern Hawaiian populations were founded from a single dispersal event. Contrary to the latter hypothesis, our mitochondrial data support a biogeographic history of multiple, relatively recent dispersals of hoary bats from North America to the Hawaiian islands. Coalescent demographic analyses of multilocus data suggest that modern populations of Hawaiian hoary bats were founded no more than 10 kya. Our finding of multiple evolutionarily significant units in Hawai'i highlights information that should be useful for re-evaluation of the conservation status of hoary bats in Hawai'i.

## Introduction

The Hawaiian islands are among the most isolated archipelagos in the world, and their native flora and fauna have served as long-standing models for island biogeography and adaptive radiation [1]. The Hawaiian archipelago has been and continues to be formed by a volcanic hotspot under the Pacific tectonic plate, and has never been connected to continental landmasses. Islands erode and increase in age as they move with the plate towards the northwest, with ages of the main Hawaiian islands ranging from 5.1–4.7 MY for the islands of Ni'ihau and Kaua'i to <0.5 MYA for the island of Hawai'i [2]. Due to their isolation, all native plant and animal species originally colonized the Hawaiian islands by long-distance dispersal via sea or air (*e*.*g*., on oceanic flotsam, passive dispersal by air currents, active flight, or indirectly hitching a ride such as seeds or snails attached to birds).

Molecular data have been key to unraveling the colonization history of several groups of terrestrial Hawaiian invertebrates and birds [3–7]. These analyses have shown that some taxa were established in the islands via multiple dispersal events from source populations outside Hawai'i, and others via a single colonization. The diversity of native Hawaiian terrestrial groups varies greatly, with some taxa being highly speciose (*e*.*g*., land snails, passerine birds, and insects), and others such as mammals being severely depauperate. With the exception of the native monk seal (*Monachus schauinslandi*), there is only one native terrestrial mammal species in Hawai'i—the hoary bat, *Lasiurus cinereus semotus*—and its origins and colonization history in the Hawaiian islands remain largely unknown.

The hoary bat, *Lasiurus cinereus sensu lato*, occupies a large continental distribution from near the latitudinal tree line in Canada, through most of North America to at least Guatemala and from Colombia to northern Argentina and Chile in South America [8]. Also, it occurs in established populations on remote oceanic island groups such as Hawai'i and the Galapagos, and extralimital records exist from locations as far as Iceland and the Orkney Islands [9–11]. No other American bat has established island populations on a similar scale.

Three subspecies of *L*. *cinereus* are currently recognized: *L*. *c*. *cinereus* in North and Central America with Philadelphia as type locality, *L*. *c*. *villosissimus* in South America with Paraguay as type locality, and *L*. *c*. *semotus* restricted to the Hawaiian islands [12]. Molecular genetic studies of *L*. *cinereus* are limited to an allozyme-based analysis of species-level variation within the genus *Lasiurus* by Baker *et al*. [13], and an analysis of mitochondrial RFLP data by Morales and Bickham [14] that supported the taxonomic distinction of North American, South American, and Hawaiian populations at the subspecific level. The latter authors included only one specimen from Hawai'i and concluded that *L*. *c*. *semotus* probably originated from North American populations, with dispersal occurring relatively recently based on low divergence values among haplotypes. A morphological study [15] documented significant divergence of Hawaiian and North American populations, and changes in wing shape and jaw mechanics that may have allowed Hawaiian hoary bats to use different habitats and prey than those of their North American counterparts. Modern sequencing tools and more robust analytical frameworks to reconstruct phylogeographic and demographic history are now available to test hypotheses regarding the number, regions of origin, and timing of *L*. *c*. *semotus* dispersal events to the Hawaiian islands.

Hoary bats have flight morphology that permits long distance dispersal and migration [16,17], including long-distance migration within the North American continent [18] and the regular colonization of oceanic islands by this species. Bonaccorso and McGuire [19] modeled energetics and water balance of simulated colonization flights for *L*. *c*. *cinereus* founders arriving in Hawai'i. They concluded that physical conditions (trade wind velocity and direction) and physiological conditions during fall migration (fat storage, energy consumption, and water balance) would allow for long distance dispersal from the Pacific coast of North America (rather than from other parts of its range), and suggested that multiple colonization events may have been possible despite the energetic and physical constraints on dispersers.

In this study we examine the phylogeography of *L*. *cinereus* from the Hawaiian islands to estimate colonization history, divergence times, and effective population sizes for Hawaiian hoary bats in the context of additional samples from North America, South America, and the Galapagos islands. We use multiple molecular markers (mitochondrial and nuclear) and analytical approaches (Bayesian and maximum likelihood) to test the following specific hypotheses:

1. Hawaiian *L*. *c*. *semotus* originated from North American *L*. *c*. *cinereus* rather than from South American *L*. *c*. *villosissimus*, and
2. Hawaiian *L*. *c*. *semotus* originated from a single colonization event.

Our study provides genetic data that can be used to guide conservation priorities for this endangered mammal, and adds to the growing body of evidence for the biogeographic origins of native Hawaiian taxa.

## Materials and Methods

### Sample collection

**Fig 1:**
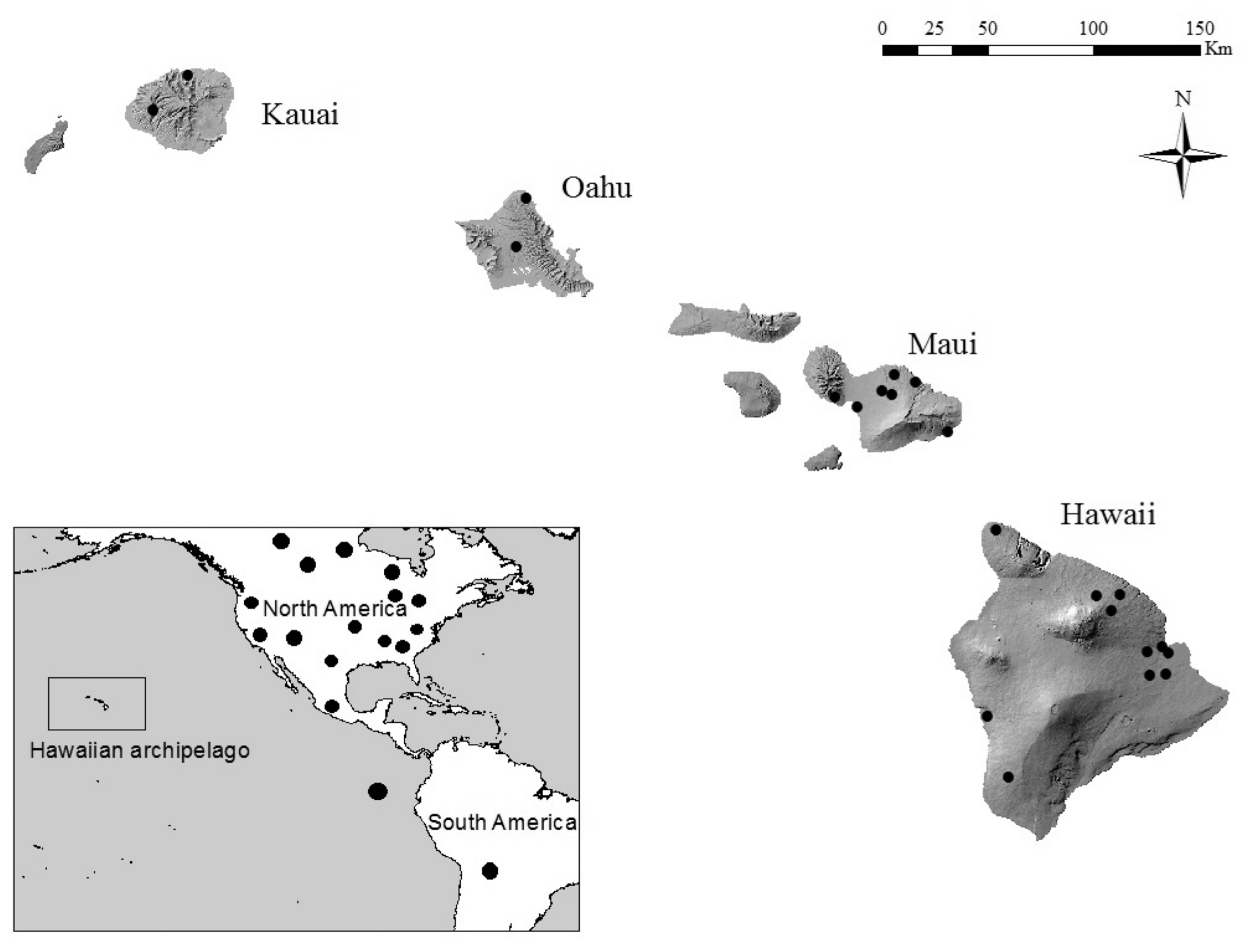
Sampling locations. The locations for *L*. *cinereus* specimens used in *CO1* analysis.

Live specimens of Hawaiian hoary bats (*Lasiurus cinereus semotus; n* = 44) were captured using mist nets in a variety of urban and forest sites on the island of Hawai'i during 2005–2012. Captured individuals were sexed, wing tissue was sampled, and bats were released on site. We used a sterile 3-mm biopsy punch to sample wing tissue [20]; tissue samples were stored in NaCl-saturated 20% DMSO or silica gel desiccant at ambient temperature in the field, and at—80°C upon return to the lab. Tissue samples from necropsied carcasses from O'ahu, Maui, and Kaua'i were donated by the U.S. Geological Survey’s National Wildlife Health Center Honolulu Field Station. These carcasses (*n* = 15) were frozen on discovery and subsequent tissue samples were stored at -80°C. Sampling locations are shown in Fig 1.

Samples of *L*. *c*. *cinereus* from continental North America (*n* = 85) represent a combination of museum tissue collections (pectoral muscle or organs stored in ethanol), mist-netted live bats, and turbine-killed bats from wind energy facilities (wing biopsies from the latter two stored in NaCl-saturated 20% DMSO). Sampling locations at the state, provincial, and island levels are provided in Table 1; more precise sampling information is available upon request.

**Table 1:** Sampling information.

Genus	Species	Subspecies	Location	Sample size (*COI*)	Sample size (*CHY*)	Sample size (*RAG2*)
*Lasiurus*	*cinereus*	*semotus*	Hawai'i	44	34	28
*Lasiurus*	*cinereus*	*semotus*	Kaua'i	2	–	–
*Lasiurus*	*cinereus*	*semotus*	Maui	9	4	8
*Lasiurus*	*cinereus*	*semotus*	O'ahu	4	–	4
*L*. *c*. *semotus* total				59	38	40
*Lasiurus*	*cinereus*	*cinereus*	Canada*	11	–	–
*Lasiurus*	*cinereus*	*cinereus*	Alberta	1	–	–
*Lasiurus*	*cinereus*	*cinereus*	Manitoba	4	–	–
*Lasiurus*	*cinereus*	*cinereus*	Ontario	2	–	–
*Lasiurus*	*cinereus*	*cinereus*	Saskatchewan	25	–	–
*Lasiurus*	*cinereus*	*cinereus*	Michoacan	2	–	–
*Lasiurus*	*cinereus*	*cinereus*	Arizona	4	–	–
*Lasiurus*	*cinereus*	*cinereus*	California	11	–	–
*Lasiurus*	*cinereus*	*cinereus*	Georgia	1	–	–
*Lasiurus*	*cinereus*	*cinereus*	Indiana	2	–	–
*Lasiurus*	*cinereus*	*cinereus*	Michigan	1	–	–
*Lasiurus*	*cinereus*	*cinereus*	Nebraska	3	–	–
*Lasiurus*	*cinereus*	*cinereus*	Tennessee	6	–	–
*Lasiurus*	*cinereus*	*cinereus*	Texas	8	–	–
*Lasiurus*	*cinereus*	*cinereus*	Washington	3	–	–
*Lasiurus*	*cinereus*	*cinereus*	West Virginia	1	–	–
*L*. *c*. *cinereus* total				85	0	0
*Lasiurus*	*cinereus*	*villosissimus*	Bolivia	1	–	–
*Lasiurus*	*cinereus*	*villosissimus*	Galapagos	1	–	–
*L*. *c*. *vilosissimus* total				2	0	0
*Lasiurus*	*intermedius*	–	–	2	–	–
*Lasiurus*	*xanthinus*	–	–	2	–	–

Sample size for the nuclear loci *CHY* and *RAG2* are given as the number of sequenced chromosomes.

* sequences from a study in which the province was not specified [21].

Wing biopsy samples from several South American *L*. *c*. *villosissimus* specimens were taken from the collection at the American Museum of Natural History. We used two specimens, from Santa Cruz, Bolivia (AMNH catalog # M-260258) and Galapagos Islands, Ecuador (AMNH catalog # M-268079), which were originally collected in 1989 and 1991, respectively. *Lasiurus intermedius* and *L*. *xanthinus* were used as outgroups for phylogenetic analyses, with samples of *L*. *intermedius* from the American Museum of Natural History (AMNH 215319) and the Angelo State Natural History Collection (ASK421 = ASNHC 1408), and of *L*. *xanthinus* from Centro de Investigaciones Biológicas del Noroestre, La Paz, Mexico.

### Ethics statement

All live animal sampling was carried out in accordance with the recommendations in the Guide for the Care and Use of Laboratory Animals of the National Institutes of Health. The protocol was approved by the Committee on the Ethics of Animal Experiments of the University of Hawai'i at Hilo (permit number 04-039-5). An Endangered Species Federal Fish and Wildlife permit (number TE003483-25) was issued by the U.S. Fish and Wildlife Service. A Protected Wildlife permit (number WL13-04) was issued by the State of Hawai'i, Department of Land and Natural Resources.

### DNA isolation and sequencing

DNA was isolated from all tissues using a DNeasy DNA isolation kit (Qiagen) according to the manufacturer’s instructions. DNA extraction from *L*. *c*. *vilosissimus* specimens used a previously described modified DNeasy protocol with extended 4 day digestion time and reduced elution volume [22]. PCR for the cytochrome c oxidase I (*COI*) gene used primers LCO1490 and HCO2198 and cycling conditions outlined by Hebert *et al*. [23]. PCR products were cleaned of excess nucleotides and primers using Exo-SAP (Affymetrix), and were sequenced at the University of Arizona Genetics Core facility, yielding a 657 bp fragment. South American specimens from the AMNH were sequenced at the Sackler Institute for Comparative Genomics core facility. Raw sequences were edited and trimmed of primer sequences using CodonCode Aligner. The mitochondrial *COI* dataset was also supplemented with sequences from GenBank [21,24]. The full dataset was aligned by eye.

Nuclear sequences from the chymase (*CHY*, 695 bp) and recombination activating gene 2 (*RAG2*, 706 bp) loci were amplified and cloned from a subset of individuals using primers from Venta *et al*. [25] and Stadelmann *et al*. [26], respectively. Comparisons of these data against the published horse genome (UCSC Genome Browser) suggest that the *CHY* locus is an EPIC marker, while the *RAG2* locus represents an exon. These loci were chosen based on pilot studies indicating consistent amplification and high variability of these markers in lasiurines (MJV). Initial PCR was conducted using illustra Hot Start mix PCR beads (GE Healthcare), with 0.5 μL of each appropriate primer and 1.0 μL of genomic DNA. Diploid amplicons were cloned using a TOPO TA cloning kit (Life Technologies) according to the manufacturer’s instructions. Five to eight colonies were picked per individual and used as template in a PCR reaction using the same primers. Reactions yielding a target-sized product were sequenced at the University of Arizona Genetics Core facility. Sequences were edited using Sequencher v.5.1 (GeneCodes) and aligned by eye. All novel sequences generated in this study have been accessioned with GenBank (accession numbers KR349974-KR350142).

### Data analysis

We used a dataset of 150 *COI* sequences to construct a phylogeny for all *L*. *cinereus* subspecies. First, redundant haplotypes were identified using Collapse v.1.2 [27] and removed, resulting in a dataset of 47 unique haplotypes (see supporting information for details on collapsed haplotypes). A maximum likelihood phylogenetic analysis was conducted using RAxML v.8 [28]. Based on the results of a jModelTest [29] analysis, we specified a GTR + Γ model, with four gamma rate categories. Support for major nodes was assessed via 1000 rapid bootstrap replicates. Resulting phylogenies were visualized using FigTree v.1.4.0, with *L*. *intermedius* designated as the outgroup. Support for alternative topologies was assessed using the approximately unbiased [30], Shimodaira-Hasegawa [31], and Kishino-Hasegawa [32] tests implemented in Consel v.0.1 [33]. To clarify relationships among haplotypes within *L*. *cinereus*, we constructed a maximum parsimony network for the mitochondrial *COI* dataset using TCS v.1.21 [34,35].

The history of population size changes in Hawaiian populations was explored using extended Bayesian skyline plots [36] in Beast v.1.7.4. These multilocus analyses used DNA sequence data from the mitochondrial *COI* locus and nuclear *CHY* and *RAG2* introns, and were conducted separately on the Hawaii1 and Hawaii2 clades identified in the maximum likelihood phylogeny (see Results). Evolutionary models for each locus and each clade were identified using jModelTest v.2.1.2 [29]. These models were approximated for the Hawaii1 clade as a TN93 model [37] with empirical nucleotide frequencies for *COI*, and an HKY model [38] with equal nucleotide frequencies for the nuclear loci. For the Hawaii2 clade, models were specified as an HKY model with empirical nucleotide frequencies for the *COI* and *RAG2* loci, and an HKY model with equal nucleotide frequencies for the *CHY* locus. Substitution rates were scaled relative to a rate of 2% per million years (My) for *COI* [39]. Substitution rate priors for nuclear loci were set to a U[0,1] distribution with an initial value of 0.02 (= 2% per My). Operator values were adjusted to a weight of 2 for all kappa values, a weight of 15 for all substitution rates and heights, a weight of 40 for the demographic.populationMeanDist variable, a weight of 100 for the demographic.indicators variable, and a weight of 60 for the demographic.scaleActive variable. Three independent runs of 20 million steps each were conducted for each population to verify consistency of results. Runs were checked for convergence by monitoring ESS values in Tracer v.1.5. Estimates of *N*_e_ were calculated assuming an average generation time of 2 years.

## Results

### Historical biogeography of hoary bats in Hawai'i

**Fig 2:**
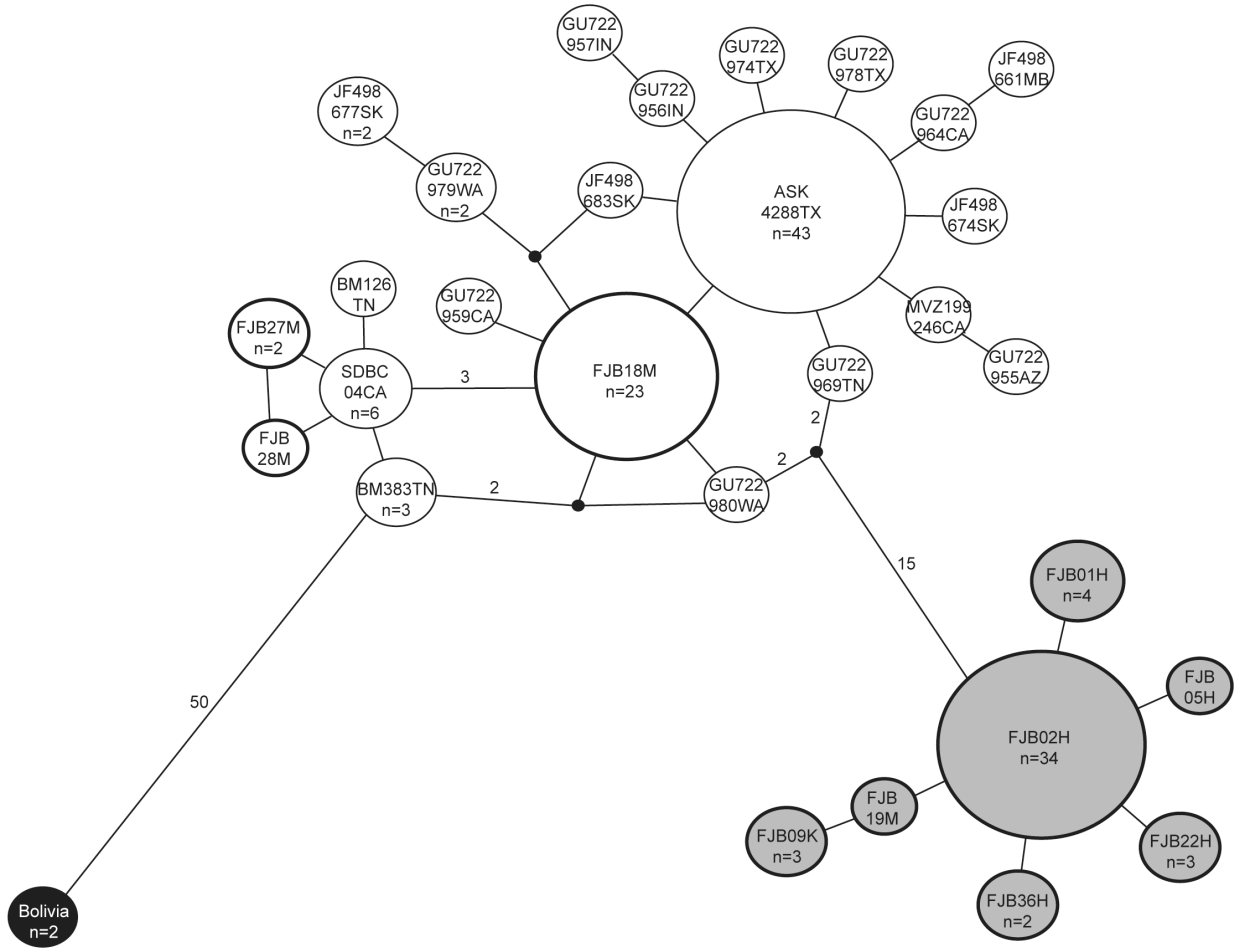
Maximum parsimony network of *CO1* haplotypes. Each haplotype is represented by a circle, the relative size of which roughly corresponds to the haplotype frequency. The number of mutations between haplotypes is indicated only for those connections spanning more than one mutation. Hawaii1 haplotypes grey, Hawaii2/North America haplotypes white, South American haplotype black. Hawaiian islands haplotypes in bold.

A maximum parsimony network was constructed for the mitochondrial dataset of 47 unique haplotypes (Fig 2). *Lasiurus cinereus* samples clearly segregated into three clusters, referred to as Hawaii1, Hawaii2/North America, and South America. Hawaiian hoary bats were divided among two clusters, one of which also included all mainland North American samples. The Hawaii1 cluster represented 48 individuals, including all 44 from the island of Hawai'i, two samples from Kaua'i, one sample from Maui, and one from O'ahu. The haplotypes in this Hawaii1 cluster were endemic to the Hawaiian islands. The Hawaii2/North America cluster included eight samples from Maui, three from O'ahu, and all those (*n* = 85) from North America. *Lasiurus cinereus semotus* samples segregating within this cluster were very closely related to *L*. *c*. *cinereus* samples; in fact, one haplotype (labelled FJB18M in Fig 2) was found on Maui and O'ahu as well as in Nebraska, Ontario, Saskatchewan, California, Georgia, and Michigan (see S1 Table). The Hawaii1 and Hawaii2/North America clusters were clearly distinct from one another (Table 2), with an average pairwise distance of 19.98 differences between them. The single haplotype detected in South America was much more distinct from the other clusters in the network, with an average distance of 55.71 and 52.45 differences between it and the Hawaii1 and Hawaii2/North America clusters, respectively.

**Table 2:** Average pairwise genetic distances (*COI*) within and between *COI* clades.

	Hawaii1	Hawaii2/NorthAmerica	South America
Hawaii1	1.90 (0.29%)	–	–
Hawaii2/NorthAmerica	19.98 (3.04%)	3.84 (0.58%)	–
South America	55.71 (8.48%)	52.45 (7.98%)	0 (0%)

A maximum likelihood analysis recovered a similar phylogenetic backbone (S1 Fig). The most likely topology described the South American subspecies *L*. *c*. *villosissimus* as sister to a clade containing both the Hawaii1 and Hawaii2/North America clades as defined in the network analysis. However, bootstrap support was low for some key internal branches, including those defining the Hawaii2/North America (75%) and Hawaii1 (70%) clades and the branch setting all *L*. *c*. *cinereus* and *L*. *c*. *semotus* samples as sister to South American *L*. *c*. *villosissimus* (60%). Topological tests (S2 Fig, S2 Table) were not able to reject any alternative topologies.

### Historical demography of Hawaiian populations

**Fig 3:**
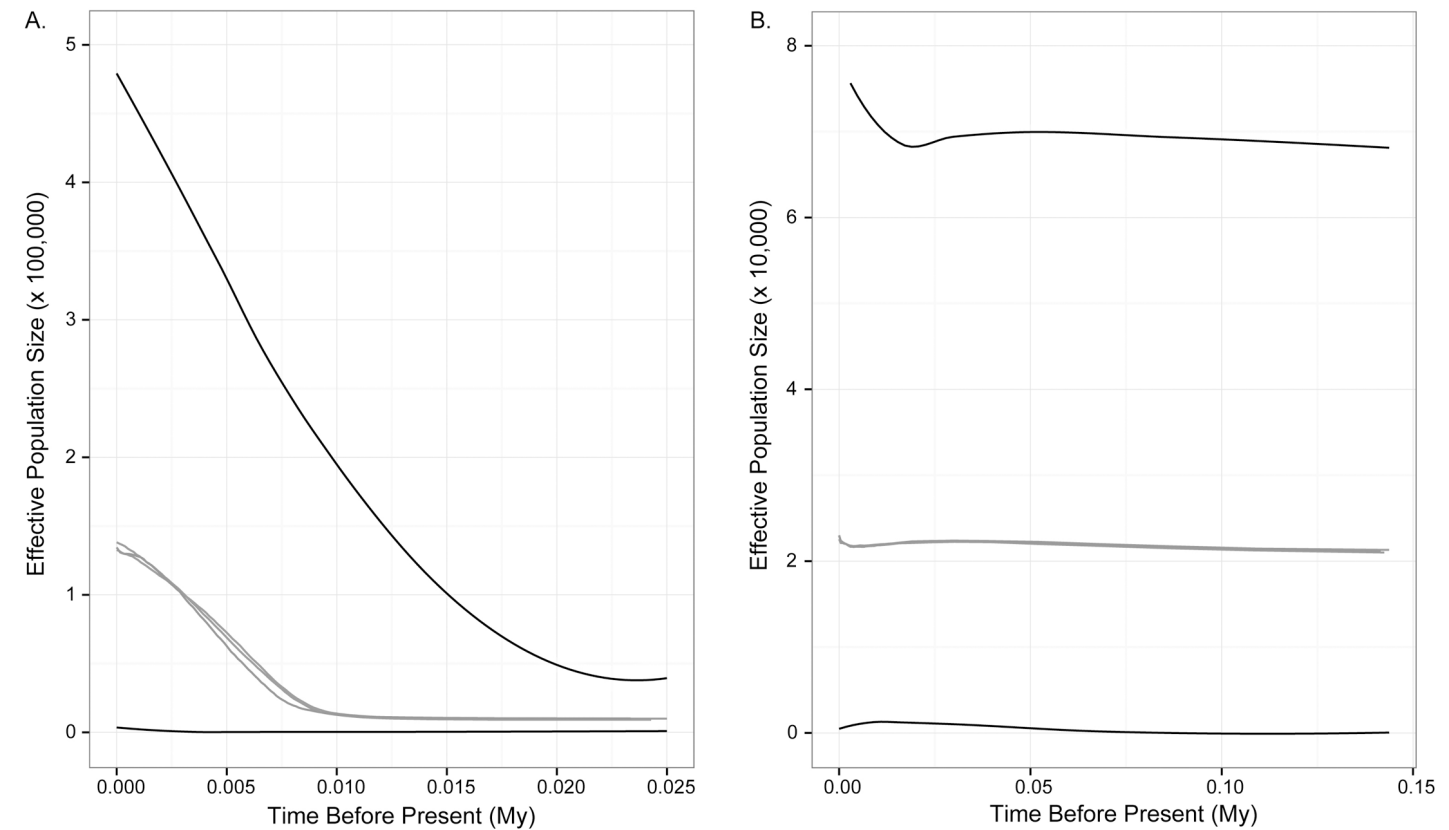
Extended Bayesian skyline plots for Hawaiian populations. Results of three runs are shown as gray lines, bounded by 95% confidence intervals in black. A. Skyline plot for the Hawaii1 population, showing a pattern of population growth starting at ca. 10 kya. B. Skyline plot for the Hawaii2 population, fitting a model of population stasis.

Extended Bayesian skyline analyses supported different demographic histories for the Hawaii1 and Hawaii2 populations of *L*. *c*. *semotus* (Fig 3). Analysis of the Hawaii1 population supported a single period of growth starting around 10 kya (Fig 3A). Ancestral effective population size prior to demographic expansion was estimated at approximately 10,000 individuals, while the current effective population size was estimated to be approximately 132,000 individuals. We emphasize that this estimate of current *N*_e_ should not be interpreted as an estimate of the present-day census population size. The Hawaii2 population, on the other hand, is consistent with a model of population stasis with an effective size of approximately 21,000 individuals (Fig 3B). There does seem to be a slight increase in the median *N*_e_ starting approximately 800 years ago. The significance of this increase is unclear, but it is a consistent pattern across replicate runs.

## Discussion

### Biogeography of Hawaiian hoary bats

We show that Hawaiian populations of hoary bats derive from at least two independent dispersal events, both originating from populations in North America. The presence in the Hawaiian archipelago of two genetically distinct lineages is indicated by both phylogenetic and parsimony network analyses of mitochondrial DNA sequence data, and is further supported by multilocus demographic analyses characterizing the distinct evolutionary histories of these two Hawaiian lineages. Previous authors have also alluded to morphological differentiation between populations on the islands of Hawai'i and Maui [40].

Our results are consistent with the physiological model of Bonaccorso and McGuire [19], who predicted that multiple colonizations of Hawai'i by North American *L*. *cinereus* were possible based on prevailing physical conditions and energetics of migrating individuals. They are also consistent with other phylogeographic studies showing that species groups of some terrestrial Hawaiian taxa were founded by multiple historic colonizations, including ducks (*Anas* sp., [41]), succineid snails [42], and katydids [43], suggesting that multiple taxa have been able to repeatedly overcome the significant challenges associated with the colonization of these remote islands.

Our results point to the colonization of Hawai'i by hoary bats on two occasions by lineages that experienced distinct evolutionary trajectories. The Hawaii1 lineage was sampled primarily from the island of Hawai'i, but was also present in low numbers on O'ahu, Maui, and Kaua'i. EBSP analyses of this lineage support a model of population growth starting around 10 kya. A single fossil specimen described from Kaua'i confirms the presence of *L*. *cinereus* in the Hawaiian islands as early as 6760 yrBP [44], consistent with our genetically-derived dates. This lineage has diverged significantly from the mainland lineage (Table 2). Given the short branch lengths within *L*. *cinereus* reconstructed in our phylogeny, we infer that the demographic expansion detected for the Hawaii1 lineage in the EBSP analysis represents population growth following dispersal to Hawai'i. This inference is also supported by the star-like topology of this clade in the haplotype network (Fig 2).

In contrast, the Hawaii2 lineage shows no significant signal of population size change. Hawaii2 mitochondrial haplotypes are not clearly differentiated from North American haplotypes, with one shared haplotype (FJB18M) found at sampling locations across North America as well as on the islands of Maui and O'ahu (S1 Table). These results suggest that the Hawaii2 lineage represents a more recent dispersal to Hawai'i.

### Future work

While we present the first examination of the colonization history and evolution of Hawaiian hoary bats, there are a number of opportunities for further research. First, the network and phylogenetic analyses were necessarily limited to the mtDNA data because most of our North American mitochondrial data were acquired via GenBank and we had access to only a limited number of tissue samples for generating new data from the continent. This also explains why our nuclear intron data were limited to Hawaiian samples. Future studies utilizing coalescent-based species tree/gene tree analyses of multilocus data [45,46] would be particularly informative for addressing taxonomic questions that are naturally raised by our study.

The fact that both the Hawaii1 and Hawaii2 lineages are found in apparent sympatry on Maui and O'ahu also requires further examination. Despite a strong sampling effort on the island of Hawai'i (*n* = 44 from 11 locations), the Hawaii2 lineage was not detected there. Because these clades are defined and differentiated solely by the mitochondrial *COI* gene, it is possible that the lack of congruent structure at the nuclear loci results from ongoing gene flow between the two populations (S3 Table). Given the recent timescales over which these dispersal events probably occurred, it is also possible that the lack of congruent structure results from incomplete lineage sorting at the nuclear loci [47]. Future work examining patterns of structure at nuclear microsatellite loci or large numbers of single nucleotide polymorophism (SNP) markers would be informative for addressing these alternatives. Furthermore, the presence of the Hawaii1 lineage and, to a lesser extent, the Hawaii2 lineage on multiple islands suggests the occurrence of significant inter-island dispersal. Microsatellite genotyping and SNP typing, along with improved sampling of the older islands, would prove useful in quantifying rates of movement among islands. Ongoing work by our research group is addressing these questions.

### Implications for hoary bat conservation

We strongly caution against the interpretation of our estimates of *N*_e_ as being indicative of the present-day census size for Hawaiian populations. If these populations were founded recently by individuals representative of the genetic diversity of the North American population, then diversity measures might well exceed that expected at mutation-drift equilibrium and our estimates of *N*_e_ would thus be much larger than the current census size [48,49]. As the founding population establishes and grows, it is possible that census size could eventually overtake effective size, but it is unclear how long that process would take.

Understanding the conservation genetics of hoary bats in Hawai'i is particularly timely and important because *L*. *c*. *semotus* is listed by the U.S. Fish and Wildlife Service [50] as Endangered, and the species recovery plan [40,51] cites molecular genetics as an area of data deficiency needed for adaptive management. Furthermore, both North American and Hawaiian hoary bats are under emerging threats from rapid expansion of the wind energy industry [52] with “take” mortalities having triggered mitigation actions in Habitat Conservation Plans for several wind facilities in Hawai'i and North America (data obtained by A. Pereira, April 2014, via a Freedom of Information Act request to the U.S. Fish and Wildlife Service).

Given the threats to the recovery of Hawaiian hoary bats, most notably those from wind energy development, barbed wire fences [53], and habitat loss [54], there is an urgent need for additional work on the conservation genetics of hoary bats to understand, among other things, their estimated effective population sizes, dispersal abilities, and genetic differentiation among and within islands. One area of importance is the resolution of potential evolutionary significant units that probably are represented by the Hawaii1 and Hawaii2 clades. The Endangered Species Act of 1973 (7 U.S.C. § 136, 16 U.S.C. § 1531 et seq.) defines species to include “any subspecies of fish or wildlife or plants, and any distinct population segment of any species of vertebrate fish or wildlife which interbreeds when mature”. Increasing field efforts to census and sample individuals from populations on islands other than the island of Hawai'i, combined with multilocus genotyping [55] of these and additional specimens should help to further clarify the taxonomic status and ESU designation of this island mammal.

## Supporting Information

S1 Fig Maximum likelihood phylogeny for sampled *L*. *cinereus* subspecies, with bootstrap support measures. Hawaiian hoary bats (*L*. *c*. *semotus*) cluster into two distinct clades, Hawaii1 and Hawaii2/North America, the latter of which is more closely related to mainland North American (*L*. *c*. *cinereus*) samples.
(TIF)

S2 Fig Constraint topologies analyzed in the topological tests. (TIF)

S1 Table Location information for redundant *COI* haplotypes used in the network analysis. (DOCX)

S2 Table Results of tests of alternative tree topologies. Significance values are provided for approximately unbiased, Kishino-Hasegawa, and Shimodaira-Hasegawa tests.
(DOCX)

S3 Table Locus-specific AMOVAs quantifying genetic structure between individuals assigned to the Hawaii1 and Hawaii2 clades. (DOCX)
