# Supplementary figures and images for: Two Tickets to Paradise: Multiple Dispersal Events in the Founding of Hoary Bat Populations in Hawai'i
Source: PLoS One. 2015 Jun 17;10(6):e0127912. doi: 10.1371/journal.pone.0127912 (PMC4471086; doi:10.1371/journal.pone.0127912)

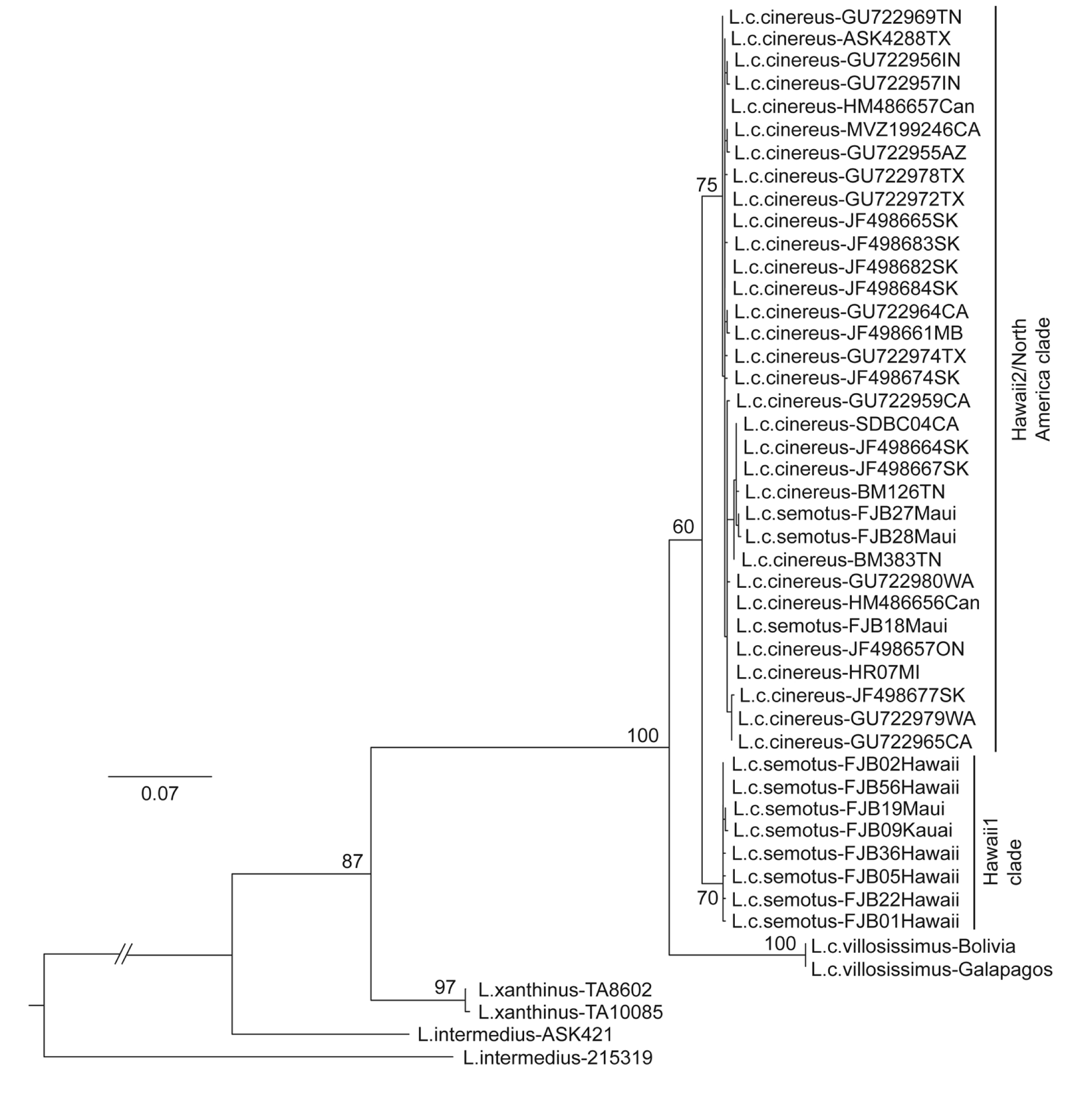

Supplement: S1 Fig — Hawaiian hoary bats (L. c. semotus) cluster into two distinct clades, Hawaii1 and Hawaii2/North America, the latter of which is more closely related to mainland North American (L. c. cinereus) samples. (TIF) [file pone.0127912.s001.tif]

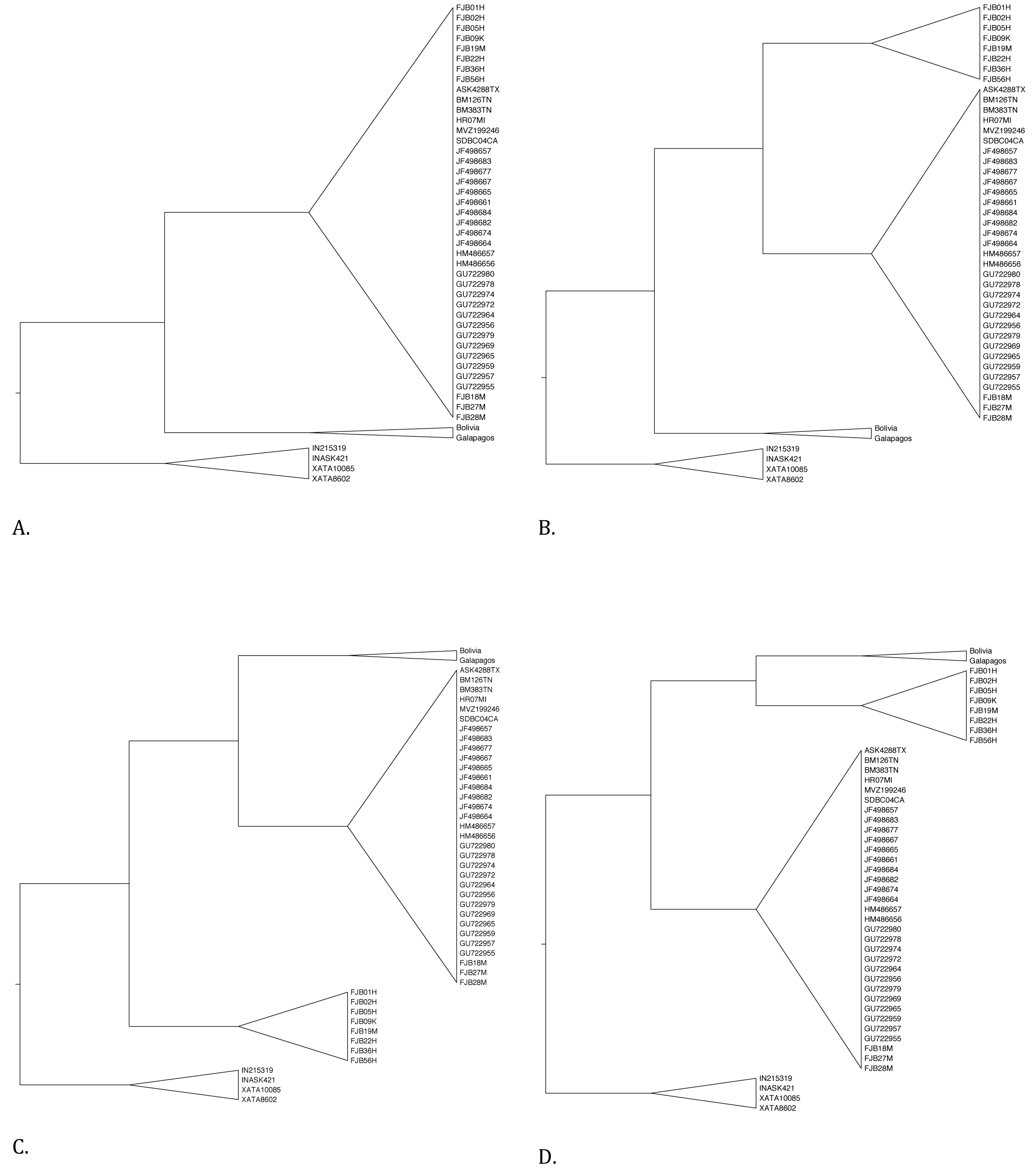

Supplement: S2 Fig — (TIF) [file pone.0127912.s002.tif]
