# Supplementary material for: Two Tickets to Paradise: Multiple Dispersal Events in the Founding of Hoary Bat Populations in Hawai'i
Source: PLoS One. 2015 Jun 17;10(6):e0127912. doi: 10.1371/journal.pone.0127912 (PMC4471086; doi:10.1371/journal.pone.0127912)
Supplement: S1 Table — (DOCX) [file pone.0127912.s003.docx]

**Supporting Information Table S1. Location information for redundant *COI* haplotypes used in the network analysis.**

| **Haplotype label** | **Taxon** | **Location of identical haplotypes** | **Haplotype copies per location** |
| --- | --- | --- | --- |
| Bolivia | *L. c. villosissimus* | Santa Cruz, Bolivia | 1 |
|  |  | Galapagos, Ecuador | 1 |
| FJB01H | *L. c. semotus* | Hawai'i, Hawai'i, USA | 4 |
| FJB02H | *L. c. semotus* | Hawai'i, Hawai'i, USA | 34 |
| FJB05H | *L. c. semotus* | Hawai'i, Hawai'i, USA | 1 |
| FJB09K | *L. c. semotus* | Kaua'i, Hawai'i, USA | 2 |
|  |  | O'ahu, Hawai'i, USA | 1 |
| FJB19M | *L. c. semotus* | Maui, Hawai'i, USA | 1 |
| FJB22H | *L. c. semotus* | Hawai'i, Hawai'i, USA | 3 |
| FJB36H | *L. c. semotus* | Hawai'i, Hawai'i, USA | 2 |
| FJB18M | *L. c. semotus* and  *L. c. cinereus* | Maui, Hawai'i, USA | 5 |
|  |  | O'ahu, Hawai'i, USA | 3 |
|  |  | Ontario, Canada | 2 |
|  |  | Saskatchewan, Canada | 6 |
|  |  | Canada | 1 |
|  |  | California, USA | 2 |
|  |  | Georgia, USA | 1 |
|  |  | Michigan, USA | 1 |
|  |  | Nebraska, USA | 2 |
| FJB27M | *L. c. semotus* | Maui, Hawai'i, USA | 2 |
| FJB28M | *L. c. semotus* | Maui, Hawai'i, USA | 1 |
| JF498661MB | *L. c. cinereus* | Manitoba, Canada | 1 |
| JF498683SK | *L. c. cinereus* | Saskatchewan, Canada | 1 |
| JF498677SK | *L. c. cinereus* | Saskatchewan, Canada | 1 |
|  |  | Texas, USA | 1 |
| JF498674SK | *L. c. cinereus* | Saskatchewan, Canada | 1 |
| GU722980WA | *L. c. cinereus* | Washington, USA | 1 |
| GU722955AZ | *L. c. cinereus* | Arizona, USA | 1 |
| MVZ199246CA | *L. c. cinereus* | California, USA | 1 |
| SDBC04CA | *L. c. cinereus* | California, USA | 1 |
|  |  | Saskatchewan, Canada | 5 |
| GU722959CA | *L. c. cinereus* | California, USA | 1 |
| GU722957IN | *L. c. cinereus* | Indiana, USA | 1 |
| BM126TN | *L. c. cinereus* | Tennessee, USA | 1 |
| BM383TN | *L. c. cinereus* | Saskatchewan, Canada | 2 |
|  |  | Tennessee, USA | 1 |
| GU722969TN | *L. c. cinereus* | Tennessee, USA | 1 |
| ASK4288TX | *L. c. cinereus* | Alberta, Canada | 1 |
|  |  | Manitoba, Canada | 3 |
|  |  | Saskatchewan, Canada | 11 |
|  |  | Canada | 10 |
|  |  | Michoacan, Mexico | 2 |
|  |  | Arizona, USA | 3 |
|  |  | California, USA | 3 |
|  |  | Washington, USA | 1 |
|  |  | Nebraska, USA | 1 |
|  |  | Tennessee, USA | 3 |
|  |  | Texas, USA | 4 |
|  |  | West Virginia, USA | 1 |
| GU722978TX | *L. c. cinereus* | Texas, USA | 1 |
| GU722974TX | *L. c. cinereus* | Texas, USA | 1 |
| GU722964CA | *L. c. cinereus* | California, USA | 1 |
| GU722956IN | *L. c. cinereus* | Indiana, USA | 1 |
| GU722979WA | *L. c. cinereus* | California, USA | 1 |
|  |  | Washington, USA | 1 |
