# Supplementary material for: Two Tickets to Paradise: Multiple Dispersal Events in the Founding of Hoary Bat Populations in Hawai'i
Source: PLoS One. 2015 Jun 17;10(6):e0127912. doi: 10.1371/journal.pone.0127912 (PMC4471086; doi:10.1371/journal.pone.0127912)
Supplement: S2 Table — Significance values are provided for approximately unbiased, Kishino-Hasegawa, and Shimodaira-Hasegawa tests. (DOCX) [file pone.0127912.s004.docx]

**Supporting Information Table S2. Results of tests of alternative tree topologies.**

| **Rank** | **Constraint topology** | **AU *P-*value** | **KH *P*-value** | **SH *P*-value** |
| --- | --- | --- | --- | --- |
| 1 | Unconstrained | 0.685 | 0.537 | 0.898 |
| 2 | Figure S2A | 0.718 | 0.463 | 0.790 |
| 3 | Figure S2B | 0.433 | 0.443 | 0.751 |
| 4 | Figure S2C | 0.408 | 0.271 | 0.272 |
| 5 | Figure S2D | 0.255 | 0.265 | 0.265 |

Significance values are provided for approximately unbiased, Kishino-Hasegawa, and Shimodaira-Hasegawa tests.
