# Supplementary material for: Two Tickets to Paradise: Multiple Dispersal Events in the Founding of Hoary Bat Populations in Hawai'i
Source: PLoS One. 2015 Jun 17;10(6):e0127912. doi: 10.1371/journal.pone.0127912 (PMC4471086; doi:10.1371/journal.pone.0127912)
Supplement: S3 Table — (DOCX) [file pone.0127912.s005.docx]

**Supporting Information Table S3. Locus-specific AMOVAs quantifying genetic structure between individuals assigned to the Hawaii1 and Hawaii2 clades.**

|  | *COI* | | *CHY* | | *RAG2* | |
| --- | --- | --- | --- | --- | --- | --- |
|  | % variation | *P*-value | % variation | *P*-value | % variation | *P*-value |
| Among populations | 95.31 | <0.0001 | -5.32 | 0.516 | 4.03 | 0.160 |
| Within populations | 4.69 | – | 105.32 | – | 95.97 | – |
